# Phthalate exposure is associated with subclinical coronary atherosclerosis: The Aragon Workers' Health Study (AWHS)

**DOI:** 10.1016/j.ajpc.2025.101072

**Published:** 2025-08-06

**Authors:** Diana María Mérida, Carolina Torrijo-Belanche, Belén Moreno-Franco, Martín Laclaustra, Jimena Rey-García, Sofía Gimeno-Ruiz, Ana Bayán-Bravo, Pilar Guallar-Castillón

**Affiliations:** a Department of Preventive Medicine and Public Health. School of Medicine, Universidad Autónoma de Madrid, 28029 Madrid, Spain; b Department of Pharmacoepidemiology and Biostatistics, Fundación Teófilo Hernando, 28290 Las Rozas de Madrid, Spain; c CIBERESP (CIBER of Epidemiology and Public Health), 28029 Madrid, Spain; d Department of Preventive Medicine and Public Health, Universidad de Zaragoza, Zaragoza, 50009 Spain; e Instituto de Investigación Sanitaria Aragón, Hospital Universitario Miguel Servet, 50009 Zaragoza, Spain; f CIBERCV (CIBER de Enfermedades Cardiovasculares), 28029, Madrid, Spain; g Department of Medicine, Psychiatry and Dermatology, Universidad de Zaragoza, 50009 Zaragoza, Spain; h Department of Internal Medicine, Hospital Universitario Rey Juan Carlos, IIS-FJD, 28933 Móstoles, Spain; i Veterinary School, Universidad de Zaragoza, 50013 Zaragoza, Spain; j Clinical Nutrition and Dietetics Unit, Department of Endocrinology and Nutrition, 12 de Octubre Hospital, 28041 Madrid, Spain; k IMDEA-Food Institute. CEI UAM+CSIC, Carretera de Cantoblanco 8, 28049 Madrid, Spain

**Keywords:** Phthalates, Endocrine disruptors, Atherosclerosis, Cardiovascular, Epidemiology

## Abstract

**Background and objectives:**

Phthalates are implicated in mechanisms underlying the development of cardiovascular disease, such as oxidative stress, lipid peroxidation, endothelial dysfunction, and activation of procoagulant pathways. This study aimed to evaluate the association between phthalate exposure and subclinical coronary atherosclerosis (SCA).

**Methods:**

A total of 1119 Spanish male workers from a car assembly plant (mean age: 50.9 years) were included. Urinary metabolites of 11 phthalates and ΣDEHP were analyzed. SCA was defined as coronary artery calcium ≥ 100 Agatston units. Logistic regression models were used to estimate the association between phthalates (natural log-transformed) and SCA, adjusting for sociodemographic, lifestyle, and cardiovascular risk factors. Dose-response relationships were explored using restricted cubic splines.

**Results:**

The prevalence of SCA was 10.2 %. Participants with SCA had a median Mono-ethyl phthalate (MEP) concentration of 126.1 µg/g-creatinine compared to 99.3 µg/g-creatinine in those without SCA. A 1-unit increase in the natural logarithm of MEP was associated with a 21 % higher prevalence of SCA (OR: 1.21; 95 % CI: 1.02–1.44), showing a linear relationship. Positive but non-significant associations were observed for MiBP and MnBP. Sensitivity analyses with phthalates in original units (ng/ml) and adjustments for urinary creatinine showed consistent results.

**Conclusions:**

Exposure to MEP is associated with an increased prevalence of SCA in male workers, suggesting that the exposure to this phthalate may play a role in the early stages of cardiovascular disease. These findings provide a basis for public health initiatives aimed at reducing phthalate exposure for the primary prevention of cardiovascular disease.

## Highlights

• This study examined the association between urinary phthalates and subclinical coronary atherosclerosis (SCA) in middle-aged male workers.
• Only mono-ethyl phthalate (MEP) was significantly associated with a higher prevalence of SCA.
• A 1-unit increase in log-transformed MEP levels was associated with a 21 % higher prevalence of SCA.
• The findings support a potential role of MEP in the early stages of cardiovascular disease.
• This study highlights the relevance of environmental exposures as modifiable cardiovascular risk factors.

## Graphical abstract

**Figure:**
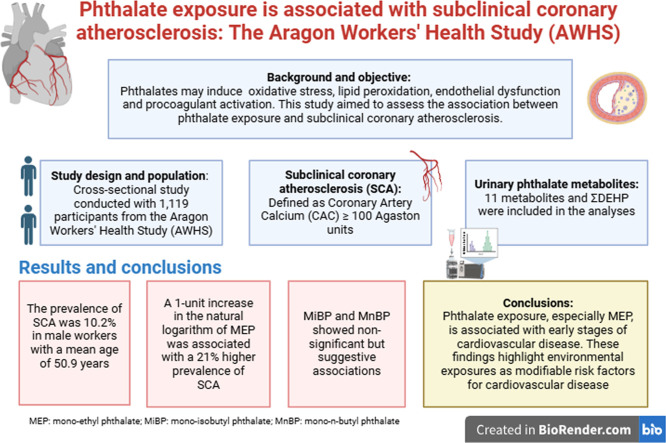

## 1 Introduction

Phthalates are ubiquitous chemicals found in a wide range of products, including food packaging and ultra-processed foods [1], personal care products, medical devices, industrial chemicals and automotive products [2]. The effects of phthalates on the cardiovascular system have been a matter of study, and it has been shown that phthalates are implicated in oxidative stress, lipid peroxidation, endothelial dysfunction and in the activation of procoagulant pathways [[3], [4], [5]], all of which play a critical role in the pathogenesis of atherosclerosis and cardiovascular disease (CVD). Comprehensive observational studies have also shown that phthalates are associated with higher prevalence of CVD [6] and higher risk of coronary heart disease (CHD) (including acute myocardial infarction and severe CHD [7]). In addition, phthalates have been associated with myocardial injury as evidenced by elevated troponin I levels [8,9].

Beyond cardiovascular outcomes, phthalates are also associated with several CVD risk factors, including obesity [10], diabetes [11], and the metabolic syndrome [12], as well as higher levels of oxidative biomarkers [9], and increased levels of fibrinogen and D-dimer [7]. Despite this scientific evidence, the association between phthalates and atherosclerosis (the most common underlying process of CVD) has been scarcely studied [[13], [14], [15], [16], [17], [18]]. However, a recent systematic review showed the relationship between phthalates and increased carotid intima-media thickness and higher prevalence of carotid plaques, which are surrogate markers of atherosclerosis [19].

Coronary atherosclerosis is characterized by plaque build-up and reduced blood flow in the vessels that perfuse the heart [20,21]. Coronary artery calcium (CAC) score provides a good estimate of atherosclerotic plaque burden [[22], [23], [24]]. Several clinical guidelines have suggested incorporating CAC score alongside risk prediction models [25], given its widespread use in predicting future cardiovascular events among asymptomatic patients [[26], [27], [28]]. Higher CAC score is associated with an increased risk of CHD [29,30], myocardial infarction, stroke and cardiovascular mortality [31]. Patients with CAC > 100 are classified as having an intermediate risk of future cardiovascular events, as it was associated with a 2.3- to 3.4-fold increase in atherosclerotic cardiovascular disease (ASCVD) and a 3.3- to 5.6-fold increased risk of CHD [30]. Also, most clinical guidelines suggest CAC > 100 as the threshold for the initiation of statins as a preventive measure for cardiovascular events [32].

In addition to traditional cardiovascular risk factors, such as hypertension, dyslipidemia, diabetes, obesity, smoking, sedentary lifestyle, and unhealthy diet [33], environmental contaminants have been identified as modifiable risk factors that are probably underestimated at present [3]. Therefore, the aim of this study was to assess the association between urinary phthalates and subclinical coronary atherosclerosis (SCA) in middle-aged male workers of a car assembly plant in Zaragoza, Spain.

## 2 Methods

### 2.1 Study population

**Fig. 1:**
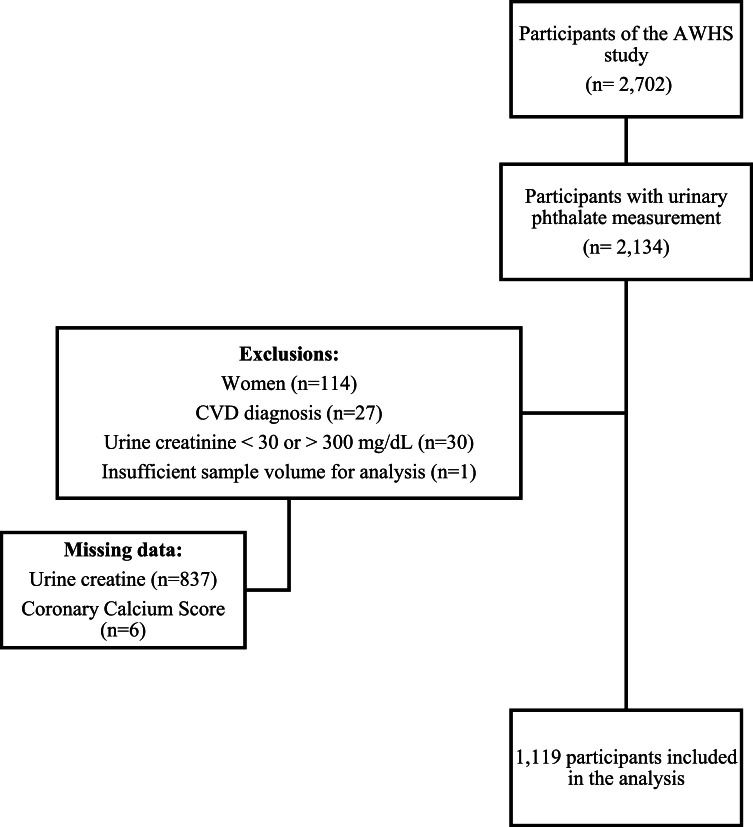
Participant flow diagram.

**Fig. 2:**
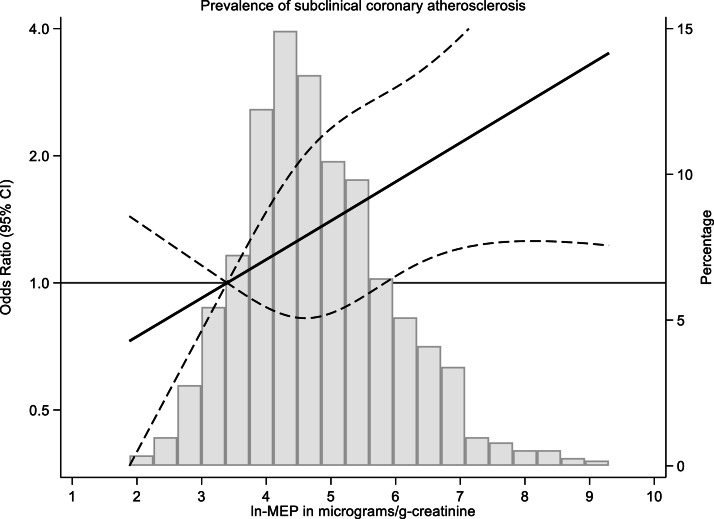
Restricted spline of the association between mono-ethyl phthalate (MEP) exposure and subclinical coronary atherosclerosis prevalence (*N* = 1119). The solid black line represents the OR of SCA across MEP levels with the 95 % CI (dashed lines). The right y-axis shows the prevalence of SCA. Abbreviations: ln-MEP: natural logarithm of MEP; CI: confidence intervals.

Cross-sectional study conducted with participants from the Aragon Workers' Health Study (AWHS). Briefly, the AWHS is a prospective cohort based on the annual health examination of workers at the General Motors Spain car assembly plant in Figueruelas (Zaragoza, Spain), recruited in 2009–2010. The design and methodology have been described elsewhere [34]. Between January 2011 and December 2014, 2167 participants aged 40–60 years underwent non-invasive imaging for subclinical atherosclerosis, completed a questionnaire interview on dietary, cardiovascular and lifestyle factors, and provided blood and urine samples after an overnight fast. Specimens were collected in polypropylene tubes and stored at − 70 °C at the Occupational Medicine Service Unit, Opel Factory, Figueruelas (Spain). Phthalates were measured in 2134 participants with available urine samples. Female participants (*n* = 114), those with insufficient sample volume for analysis (*n* = 1), previous diagnosis of CVD (*n* = 27), urinary creatinine < 30 mg/dL or > 300 mg/dL (*n* = 30) (cut-off points established by the World Health Organization to identify highly dilute or concentrated samples [35]), missing data on urinary creatinine (*n* = 837) or CAC (*n* = 6) were excluded. Finally, 1119 participants were included in the data analysis (Fig. 1, Fig. 2). All participants gave written informed consent, and the study was approved by the Central Institutional Review Board of Aragón (CEICA).

### 2.2 Coronary artery calcium

CAC was determined using non-contrast ECG-gated prospective acquisition with a multidetector computed tomography (CT) scanner (Mx 8000 IDT 16, Philips Medical System, Best, The Netherlands). CAC was quantified according to the Agatston method [36], which is the sum of the attenuation value (in Hounsfield units) and the area of all CAC lesions in the coronary arteries [37]. The CAC has been used as a proxy for the SCA [31]. For the purpose of the study and to identify those participants with clinically meaningful subclinical atherosclerosis, we defined SCA as CAC ≥ 100. This threshold is associated with increased risk of ASCVD and CHD and is frequently used in clinical practice to guide preventive interventions such as statin initiation [28,32].

### 2.3 Phthalates

The preparation of the urine samples is described elsewhere [38]. Phthalates were measured by liquid chromatography coupled to tandem mass spectrometry (LC-MS/MS), using electrospray ionization in negative mode. The following 14 urinary phthalate metabolites were measured: Mono-ethyl phthalate (MEP), Mono-isobutyl phthalate (MiBP), Mono-n‑butyl phthalate (MnBP), Monobenzyl phthalate (MBzP), Mono(2-ethyl-5-oxohexyl) phthalate (MEOHP), Mono(2-ethyl-5-carboxypentyl) phthalate (MECPP), Mono(2-ethyl-5-hydroxyhexyl) phthalate (MEHHP), Mono(2-carboxymethylhexyl) phthalate (MCMHP), Mono-cyclohexyl phthalate (MCHP), Mono-n-pentyl phthalate (MnPeP), Mono‑hydroxy‑isononyl phthalate (OH-MiNP), Mono-carboxy-isodecyl phthalate (cx-MiDP), Mono‑hydroxy‑isodecyl phthalate (OH-MiDP), and Mono-n-octyl phthalate (MnOP). The molar sum of Di(2-ethylhexyl) phthalate (ΣDEHP) was also calculated by dividing each DEHP metabolite (ng/ml) by its molar weight (g/mol) and then summing the results: [(MEOHP/292.33) + (MECPP/308.33) + (MEHHP/294.34) + (MCMHP/308.33)] [39]. Phthalate parent compounds, molecular weight, and CAS registry number are described in Table S1.

The limit of quantification (LoQ) was 0.50 ng/ml for MEP, MBzP, MEOHP, MECPP, MEHHP, MCMHP, MCHP, and MnPeP; and 1.00 ng/ml for MiBP, MnBP, OH-MiNP, cx-MiDP, OH-MiDP, and MnOP. Phthalate metabolites with > 15 % values below the LoQ were excluded. Values below the LoQ were replaced by LoQ/√2 [40]. Finally, 11 metabolites and ΣDEHP were included in the analyses.

To account for urinary dilution, phthalate concentrations (ng/ml) were divided by urinary creatinine (mg/dL) and multiplied by 100 to obtain urinary metabolite concentrations in µg/g creatinine [41].

### 2.4 Covariates

The age of the workers at the time of the physical examination was considered. Body mass index (BMI) was calculated as weight (kg) divided by height (m) squared and further classified as normal (< 25 kg/m^2^), overweight (≥25 - <30 kg/m^2^), and obesity (≥30 kg/m^2^). Smoking status was self-reported as non-smoker, former smoker, and current smoker. Alcohol consumption (grams/day) was obtained using a 136-item food frequency questionnaire (FFQ) validated in Spain [[42], [43], [44]]. Work type was categorized as office and manual work. There were two rotating shifts: morning/afternoon (06:00–14:00 and 14:00–22:00) and morning/afternoon/night (06:00–14:00, 14:00–22:00 and 22:00–06:00), and two fixed shifts: central (08:00–16:00) and night (22:00–06:00). Hypertension (HTN) was defined as systolic blood pressure (SBP) ≥ 140 mmHg or diastolic blood pressure (DBP) ≥ 90 mmHg or use of antihypertensive treatment [45]. Sample collection and laboratory methods are described elsewhere [34]. Briefly, dyslipidemia was defined as total cholesterol (TC) ≥ 240 mg/dL or LDL ≥ 160 mg/dL or HDL < 40 mg/dL or triglycerides (TG) ≥ 150 mg/dL or use of lipid-lowering treatment [46]. LDL was calculated using the Friedewald equation if TG <400 mg/dL, otherwise Sampson equation was used [47]. Diabetes Mellitus (DM) was defined as fasting glucose ≥ 126 mg/dL or use of antidiabetic treatment [48]. Physical activity (METs-h/week) was assessed using a validated version of the Spanish Nurses’ Health Study and the Health Professionals’ Follow-up Study [49]. Total energy intake (Kcal/day) derived from the Spanish food composition tables was considered as a covariate [50,51]. In sensitivity analyses, urinary creatinine (mg/dL) was included as a covariate to adjust for urinary dilution.

### 2.5 Statistical analysis

The phthalates in µg/g-creatinine were transformed to the natural logarithm (ln) and were also categorized into quartiles (the first quartile was used as a reference). To assess the association between phthalates and SCA, logistic regression models were used providing the odds ratio (OR) for SCA (CAC ≥ 100 versus CAC < 100) and the corresponding 95 % confidence intervals (CI). Two logistic regression models (crude and adjusted) were fitted analyzing both as continuous variable (ln transformed) and as quartiles. The adjusted model accounted for age (continuous), BMI (<25, ≥25 - <30, ≥30), smoking status (non-smoker, former, current), alcohol consumption (g/day), work type (office/manual work), work turn (morning/afternoon, morning/afternoon/night, central and night), hypertension (no/yes), dyslipidemia (no/yes), diabetes (no/yes), physical activity (METs-h/week), and total energy intake (Kcal/day). P for linear trend was calculated including quartiles as continuous variables in the models.

Two sensitivity analyses were performed. First, phthalates were analyzed in ng/ml and adjusted for urine creatinine in the regression models. Second, the association was analyzed after excluding participants with diabetes (*n* = 60), and the model was further adjusted for fasting glucose levels as a continuous covariate.

Restricted cubic spline regression with three knots (10th, 50th, and 90th percentile) was also fitted to evaluate the dose-response relationship between phthalates (ln-transformed) and SCA (dichotomous). As an additional statistical approach to better understand the linear relationship between phthalate exposure and CAC, this association was also evaluated using linear regression models adjusted for covariates. Since CAC did not follow a normal distribution, values were log-transformed as ln(CAC + 1), given that the logarithm of zero is undefined. Results were displayed using predicted margins plots, illustrating adjusted values of ln(CAC+1) and their 95 % CI across the range of ln(phthalate) concentrations. Analyses were performed using Stata SE 17, and statistical significance was set at *p*-value < 0.05 (two-tail).

## 3 Results

### 3.1 Characteristics of the participants

The study included 1119 participants with a mean age of 50.9 years. Of the total participants, 79 % were overweight or obese, former smokers (43.3 %), manual workers (87.6 %), and working the morning/afternoon shift (61.5 %). Regarding chronic diseases, 39.3 % had hypertension, 65.2 % had dyslipidemia, and only 5.4 % had diabetes. Regarding lifestyle factors, participants had a mean alcohol intake of 22.5 g/day, an average physical activity level of 35.4 METs-h/week, and a total energy intake of 2948 kcal/day. A total of 114 cases of SCA (CAC ≥ 100) were observed (10.2 %). No differences were found in the distribution of covariates across quartiles of MEP exposure, except for diabetes. Participants in the highest quartile of MEP exposure had twice as many cases of diabetes as those in the lowest quartile (Table 1).

**Table 1:** Baseline characteristics according to quartiles of mono-ethyl phthalate exposure (*N* = 1119).

	Mono-ethyl phthalate (MEP) (µg/g-creatinine)					
	Total participants (*N* = 1119)	Q1 (lowest)	Q2	Q3	Q4 (highest)	p-for linear trend
Age, mean ± SD	50.9 ± 3.67	50.7 ± 3.87	50.7 ± 3.44	51.0 ± 3.66	51.1 ± 3.68	0.084
BMI, n (%)						
< 25 kg/m^2^	235 (21.0)	55 (19.7)	54 (19.3)	57 (20.4)	69 (24.7)	0.180
25–29 kg/m^2^	639 (57.1)	160 (57.1)	170 (60.7)	169 (60.4)	140 (50.2)	
≥ 30 kg/m^2^	245 (21.9)	65 (23.2)	56 (20.0)	54 (19.3)	70 (25.1)	
Physical activity (METs-h/week), mean ± SD	35.4 ± 21.5	35.2 ± 20.7	34.1 ± 19.9	36.3 ± 23.2	36.0 ± 22.1	0.430
Total energy (Kcal/day), mean ± SD	2948 ± 769	2944 ± 756	2908 ± 757	2988 ± 792	2952 ± 773	0.614
Alcohol (g/day), mean (SD)	22.5 ± 21.3	21.6 ± 21.4	20.7 ± 20.9	23.9 ± 21.8	23.7 ± 21.1	0.092
Smoking, n (%)						
Non-smoker	253 (22.6)	75 (26.8)	58 (20.7)	61 (21.8)	59 (21.2)	0.602
Former smoker	485 (43.3)	113 (4.4)	127 (45.4)	126 (45.0)	119 (42.7)	
Current smoker	381 (34.1)	92 (32.9)	95 (33.9)	93 (33.2)	101 (36.2)	
Hypertension, n (%)	440 (39.3)	108 (38.6)	111 (39.6)	109 (38.9)	112 (40.1)	0.760
Dyslipidemia, n (%)	730 (65.2)	172 (61.4)	185 (66.1)	182 (65.0)	191 (68.5)	0.116
Diabetes, n (%)	60 (5.36)	9 (3.21)	14 (5.00)	17 (6.07)	20 (7.17)	0.033*
Work type, n (%)						
Manual work	980 (87.6)	245 (87.5)	243 (86.8)	248 (88.6)	244 (87.5)	0.851
Work shift, n (%)						
Morning/afternoon	688 (61.5)	163 (58.2)	174 (62.1)	177 (63.2)	174 (62.4)	0.914
Morning/afternoon/night	241 (21.5)	66 (23.6)	58 (20.7)	60 (21.4)	57 (20.4)	
Central	94 (8.40)	28 (10.0)	22 (7.86)	23 (8.21)	21 (7.53)	
Night	96 (8.58)	23 (8.21)	26 (9.29)	20 (7.14)	27 (9.68)	
Urinary creatinine (mg/dL), mean ± SD	154.5 ± 57.2	155.3 ± 57.1	152.2 ± 54.7	158.7 ± 59.3	151.8 ± 57.6	0.795
Glucose (mg/dL), mean ± SD	99.0 ± 17.5	97.6 ± 17.3	99.7 ± 20.8	98.9 ± 14.6	99.8 ± 16.8	0.230
Subclinical coronary atherosclerosis, n (%)	114 (10.2)	23 (8.21)	26 (9.29)	30 (10.71)	35 (12.54)	0.359

⁎ *p* < 0.05 BMI: body mass index; CAC: coronary artery calcium; METs: metabolic equivalent of task; SD: standard deviation. Working shifts: morning/afternoon (06:00–14:00 and 14:00–22:00), morning/afternoon/night (06:00–14:00, 14:00–22:00 and 22:00–06:00), central (08:00–16:00) and night (22:00–06:00). Quartiles of MEP exposure: Q1 (6.58–54.55), Q2 (54.61–101.37), Q3 (101.56–245.68), Q4 (246.11–10,925.80).

The phthalate with the highest median concentration was MEP, followed by MiBP and MnBP (Table 2). Participants with SCA (CAC ≥ 100) had a median MEP concentration of 126.1 µg/g-creatinine compared to a median of 99.3 µg/g-creatinine in those without SCA (CAC < 100). The remaining phthalates did not show statistically significant differences between the CAC groups (Table S2).

**Table 2:** Urinary phthalate metabolites distribution (*N* = 1119).

	LoQ	< LoQ	Phthalate (ng/ml)					Phthalate/creatinine (µg/g-creatinine)*				
			Min	Max	P25	P50	P75	Min	Max	P25	P50	P75
**MEP**	0.50	0	5.07	18,060.34	74.78	149.67	379.52	6.58	10,925.79	54.55	101.37	245.68
**MiBP**	1.00	0	2.43	1763.02	14.93	24.45	38.14	3.59	897.67	11.58	16.31	23.70
**MnBP**	1.00	0	1.94	17,620.50	12.47	19.97	31.27	2.61	14,478.64	8.90	13.56	19.66
**MBzP**	0.50	2	0.56	273.53	4.90	7.97	13.67	0.41	162.48	3.47	5.45	8.91
**MEOHP**	0.50	0	0.50	999.14	6.02	9.70	15.73	0.58	406.65	4.28	6.40	9.94
**MECPP**	0.50	0	1.12	883.88	11.79	19.73	31.65	1.29	449.15	8.68	13.01	20.11
**MEHHP**	0.50	0	1.01	1298.79	11.10	18.60	31.01	1.16	585.84	8.05	12.24	19.17
**MCMHP**	0.50	3	0.50	159.68	2.68	4.22	6.48	0.38	66.56	1.98	2.79	4.05
**MCHP**	0.50	1106	0.51	50.83	0.58	0.76	1.38	NA	NA	NA	NA	NA
**MnPEP**	0.50	1116	0.59	0.96	0.59	0.67	0.96	NA	NA	NA	NA	NA
**OH-MiNP**	1.00	0	1.01	999.95	7.03	12.01	21.54	1.06	1340.41	5.01	8.17	14.64
**cx-MiDP**	1.00	142	1.00	136.83	1.55	2.18	3.37	0.26	148.73	0.93	1.30	2.05
**OH-MiDP**	1.00	147	1.00	128.89	1.76	2.71	4.29	0.26	96.04	1.03	1.61	2.60
**MnOP**	1.00	1117	2.01	2.44	2.01	2.23	2.44	NA	NA	NA	NA	NA
**ΣDEHP (nmol/ml)**	NA	NA	0.01	11.21	0.11	0.18	0.28	0.01⁎⁎	4.56⁎⁎	0.08⁎⁎	0.12⁎⁎	0.17⁎⁎

Phthalate abbreviations: MEP: mono-ethyl phthalate; MiBP: mono-isobutyl phthalate; MnBP: mono-n‑butyl phthalate; MBzP: monobenzyl phthalate; MEOHP: mono(2-ethyl-5-oxohexyl) phthalate; MECPP: mono(2-ethyl-5-carboxypentyl) phthalate; MEHHP: mono(2-ethyl-5-hydroxyhexyl) phthalate; MCMHP: mono(2-carboxymethylhexyl) phthalate; MCHP: mono-cyclohexyl phthalate; MnPeP: mono-n-pentyl phthalate; OH-MiNP: mono‑hydroxy‑isononyl phthalate; cx-MiDP: mono-carboxy-isodecyl phthalate, OH-MiDP: mono‑hydroxy‑isodecyl phthalate, MnOP: mono-n-octyl phthalate; ΣDEHP: molar sum of di(2-ethylhexyl) phthalate.

LoQ: limit of quantification; NA: not applicable.

⁎ Phthalate metabolites with > 15 % values below the LoQ were excluded. Values below the LoQ were replaced by LoQ/√2.

⁎⁎ ΣDEHP concentrations in µmol/g-creatinine.

### 3.2 Association between phthalates and SCA

**Fig. 3:**
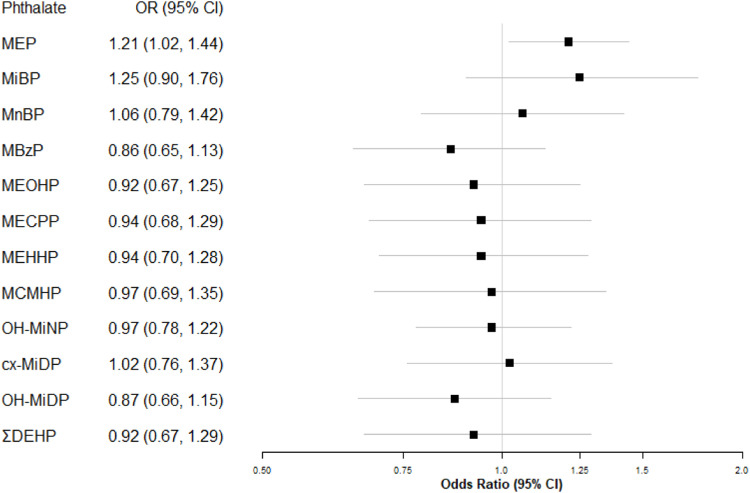
Plot of Odds Ratios and 95 % confidence intervals for the association between phthalates (per 1-unit increase in natural logarithm) and subclinical coronary atherosclerosis. Results correspond to the adjusted model, controlling for age, BMI, smoking status, alcohol consumption, work type, work turn, hypertension, dyslipidemia, diabetes, physical activity, and total energy intake (as shown in Table 3). Abbreviations: OR: Odds Ratio; CI: confidence interval.

After adjustment for potential confounders, a 1-unit increase in the natural logarithm of MEP was associated with a 21 % higher prevalence of SCA (OR = 1.21, 95 % CI: 1.02–1.44) (Table 3). The restricted cubic spline confirmed this linear trend: the higher the MEP exposure, the higher the prevalence of SCA. This association reached statistical significance when MEP concentrations were above 6 in the ln-MEP in µg/g-creatinine (Fig. 2). A non-significant higher prevalence of SCA was observed when comparing the extreme quartiles of MEP (Table 3). The association between MiBP and MnBP with SCA followed the same direction (OR > 1) as MEP without reaching statistical significance (Fig. 3).

**Table 3:** Association between phthalates and subclinical coronary atherosclerosis (*N* = 1119).

	Phthalates as continuous		Phthalates in quartiles				
Phthalate metabolite (µg/g-creatinine)	OR (95 % CI) for 1-unit increase in natural log	p-value	Q1 OR (95 % CI)	Q2 OR (95 % CI)	Q3 OR (95 % CI)	Q4 OR (95 % CI)	p-for linear trend
MEP							
Events/n			23/280	26/280	30/280	35/279	
Crude model	1.24 (1.05–1.45)⁎⁎	0.009	1 (Ref.)	1.14 (0.64–2.06)	1.34 (0.76–2.37)	1.60 (0.92–2.79)	0.076
Adjusted model	1.21 (1.02–1.44)*	0.029	1 (Ref.)	1.08 (0.58–1.99)	1.18 (0.64–2.14)	1.37 (0.76–2.46)	0.270
MiBP							
Events/n			22/280	30/280	29/280	33/279	
Crude model	1.20 (0.88–1.64)	0.237	1 (Ref.)	1.40 (0.79–2.51)	1.35 (0.76–2.42)	1.57 (0.89–2.77)	0.154
Adjusted model	1.25 (0.90–1.76)	0.181	1 (Ref.)	1.52 (0.82–2.79)	1.31 (0.71–2.43)	1.52 (0.83–2.78)	0.264
MnBP							
Events/n			18/280	33/280	32/280	31/279	
Crude model	1.12 (0.87–1.45)	0.379	1 (Ref.)	1.94 (1.07–3.54)*	1.88 (1.03–3.43)*	1.82 (0.99–3.34)	0.091
Adjusted model	1.06 (0.79–1.42)	0.715	1 (Ref.)	1.91 (1.01–3.58)*	1.73 (0.91–3.26)	1.58 (0.83–2.99)	0.284
MBzP							
Events/n			32/280	22/280	35/280	25/279	
Crude model	0.87 (0.67–1.12)	0.275	1 (Ref.)	0.66 (0.37–1.17)	1.11 (0.66–1.85)	0.76 (0.44–1.32)	0.734
Adjusted model	0.86 (0.65–1.13)	0.269	1 (Ref.)	0.63 (0.34–1.15)	1.22 (0.71–2.10)	0.76 (0.42–1.38)	0.876
MEOHP							
Events/n			25/280	32/280	33/280	24/279	
Crude model	0.91 (0.69–1.20)	0.504	1 (Ref.)	1.32 (0.76–2.28)	1.36 (0.79–2.36)	0.96 (0.53–1.73)	0.940
Adjusted model	0.92 (0.67–1.25)	0.581	1 (Ref.)	1.27 (0.70–2.28)	1.28 (0.71–2.29)	1.00 (0.54–1.85)	0.999
MECPP							
Events/n			21/280	35/280	36/280	22/279	
Crude model	0.95 (0.71–1.27)	0.728	1 (Ref.)	1.76 (1.00–3.11)	1.82 (1.03–3.20)	1.06 (0.57–1.97)	0.849
Adjusted model	0.94 (0.68–1.29)	0.690	1 (Ref.)	1.83 (1.00–3.34)*	1.90 (1.04–3.47)*	1.04 (0.54–2.01)	0.893
MEHHP							
Events/n			28/280	30/280	29/280	27/279	
Crude model	0.94 (0.72–1.24)	0.664	1 (Ref.)	1.08 (0.63–1.86)	1.04 (0.60–1.80)	0.96 (0.55–1.68)	0.870
Adjusted model	0.94 (0.70–1.28)	0.709	1 (Ref.)	0.94 (0.53–1.68)	0.91 (0.51–1.63)	0.99 (0.55–1.80)	0.955
MCMHP							
Events/n			31/280	27/280	29/280	27/279	
Crude model	0.89 (0.65–1.22)	0.468	1 (Ref.)	0.86 (0.50–1.48)	0.92 (0.54–1.59)	0.86 (0.50–1.48)	0.668
Adjusted model	0.97 (0.69–1.35)	0.844	1 (Ref.)	0.98 (0.55–1.76)	1.08 (0.60–1.91)	0.95 (0.53–1.70)	0.938
OH-MiNP							
Events/n			30/280	28/280	26/280	30/279	
Crude model	1.03 (0.84–1.27)	0.780	1 (Ref.)	0.93 (0.54–1.60)	0.85 (0.49–1.48)	1.00 (0.59–1.71)	0.940
Adjusted model	0.97 (0.78–1.22)	0.814	1 (Ref.)	0.95 (0.53–1.70)	0.60 (0.33–1.10)	0.90 (0.51–1.59)	0.436
cx-MiDP							
Events/n			25/281	30/279	32/280	27/279	
Crude model	1.04 (0.80–1.36)	0.756	1 (Ref.)	1.23 (0.71–2.16)	1.32 (0.76–2.29)	1.10 (0.62–1.94)	0.707
Adjusted model	1.02 (0.76–1.37)	0.895	1 (Ref.)	1.09 (0.60–1.96)	1.18 (0.66–2.11)	0.99 (0.54–1.82)	0.947
OH-MiDP							
Events/n			34/280	23/280	29/280	28/279	
Crude model	0.92 (0.71–1.19)	0.539	1 (Ref.)	0.65 (0.37–1.13)	0.84 (0.49–1.41)	0.81 (0.47–1.37)	0.605
Adjusted model	0.87 (0.66–1.15)	0.321	1 (Ref.)	0.61 (0.34–1.09)	0.76 (0.44–1.33)	0.69 (0.39–1.22)	0.310
ΣDEHP (µmol/g-creatinine)							
Events/n			26/280	26/280	38/280	24/279	
Crude model	0.93 (0.69–1.25)	0.637	1 (Ref.)	1.00 (0.57–1.77)	1.53 (0.90–2.60)	0.92 (0.51–1.65)	0.780
Adjusted model	0.92 (0.67–1.29)	0.657	1 (Ref.)	1.03 (0.56–1.88)	1.42 (0.80–2.50)	0.96 (0.52–1.78)	0.785

⁎ *p* < 0.05.

⁎⁎ *p* < 0.01 OR: Odds Ratio; CI: confidence intervals. Phthalate abbreviations: MEP: mono-ethyl phthalate; MiBP: mono-isobutyl phthalate; MnBP: mono-n‑butyl phthalate; MBzP: monobenzyl phthalate; MEOHP: mono(2-ethyl-5-oxohexyl) phthalate; MECPP: mono(2-ethyl-5-carboxypentyl) phthalate; MEHHP: mono(2-ethyl-5-hydroxyhexyl) phthalate; MCMHP: mono(2-carboxymethylhexyl) phthalate; MCHP: mono-cyclohexyl phthalate; MnPeP: mono-n-pentyl phthalate; OH-MiNP: mono‑hydroxy‑isononyl phthalate; cx-MiDP: mono-carboxy-isodecyl phthalate, OH-MiDP: mono‑hydroxy‑isodecyl phthalate, MnOP: mono-n-octyl phthalate; ΣDEHP: molar sum of di(2-ethylhexyl) phthalate. Crude model: unadjusted. Adjusted model: age (continuous), BMI (<25, ≥25 - <30, ≥30), smoking status (non-smoker, former, current), alcohol consumption (g/day), work type (office/manual work), work turn (morning/afternoon, morning/afternoon/night, central and night), hypertension (no/yes), dyslipidemia (no/yes), diabetes (no/yes), physical activity (METs-h/week), total energy intake (Kcal/day).

To further explore the dose–response relationship between phthalates and CAC, we conducted linear regression analyses using ln(CAC+1) as the outcome. Predicted margins plots illustrated a modest linear increase in ln(CAC+1) with higher ln(MEP) levels (Figure S1), consistent with the logistic regression findings (Fig. 2, Fig. 3). Similar positive trends were observed for MiBP, MnBP, and OH-MiNP, though with wider 95 % CIs and flatter slopes. In contrast, negative trends were noted for MBzP, MEHHP, and MCMHP. Overall, these results aligned with the main analyses.

In the sensitivity analyses, results were consistent with the main findings when phthalates were analyzed in their original units (ng/ml) with additional adjustment for urinary creatinine (Table S3). Results were also robust when participants with diabetes were excluded from the models, with further adjustment for fasting glucose levels as a continuous covariate (Table S4).

## 4 Discussion

In this well-characterized sample of male workers, a 1-unit increase in the natural logarithm of MEP exposure was associated with a 21 % higher prevalence of SCA (CAC ≥ 100), independent of sociodemographic, lifestyle, and CVD risk factors. A total of 114 cases of SCA (10.2 %) were identified, with participants in the highest quartile of MEP exposure having a median concentration of 126.1 µg/g creatinine compared with 99.3 µg/g creatinine in those without SCA. Positive but non-significant associations were observed for MiBP and MnBP. These results highlight a linear relationship between MEP exposure and SCA prevalence, which was visualized using a restricted cubic spline and showed positive significant associations at higher MEP concentrations.

Our findings are consistent with previous research showing that MEP exposure was associated with a 54 % higher prevalence of carotid plaques among community-dwelling older adults in Sweden, when comparing extreme quintiles of exposure [13]. Similarly, a study conducted among type 2 diabetic patients in China found that MEP was associated with a 14 % higher prevalence of self-reported CVD, including CHD, myocardial infarction, and stroke [17]. Taken together, these studies suggest that MEP is positively associated with early stages of CVD and major cardiovascular events. However, it is important to emphasize that all the available evidence (three studies including ours) is based on cross-sectional designs which limit the ability to establish causality.

Diethyl phthalate (DEP), the parent compound of MEP, is widely used as a plasticizer [52] (chemicals used to make plastic softer and more flexible) in various industries and products, including perfumes, mosquito repellents, toothbrushes, automotive parts, tools, toys, and food packaging [53]. While the primary concern regarding DEP has been environmental [54], it is also being evaluated as an endocrine disruptor (substances that disrupt the synthesis, metabolism, and function of hormones) [[55], [56], [57]]. Our study population includes male workers in a car assembly plant who may be exposed to some products containing DEP. Therefore, occupational exposure to this chemical could be considered as a potential cardiovascular risk factor in this subgroup of the population, especially given that occupational exposure to DEP has been a matter of interest [[58], [59], [60], [61]]. The cardiovascular effects of DEP should be investigated for public health purposes and the regulation of DEP could potentially be a measure for the primary prevention of CVD among workers. It is noteworthy that although several phthalates have been restricted in specific products [[62], [63], [64]], DEP has only been restricted in food contact materials [65].

The association between MEP exposure and higher coronary calcification may be explained by several biological mechanisms, particularly those involving the pro-atherogenic and pro-senescence effects of MEP. MEP has been shown to induce oxidative stress and disrupt lipid metabolism, promoting the degradation, oxidation, and aggregation of both HDL and LDL cholesterol. These processes impair the protective role of HDL and enhance the atherogenic properties of LDL, leading to increased foam cell formation through accelerated LDL phagocytosis by macrophages [4]. Animal studies have associated MEP exposure with significant metabolic disturbances, including the accumulation of cholesterol and triglycerides in the liver, which may contribute to systemic dyslipidemia [66]. Furthermore, MEP has been shown to induce endothelial dysfunction by reducing nitric oxide bioavailability and promoting vascular inflammation [67]. These combined effects suggest that MEP exposure may create a pro-atherogenic environment conducive to the development and progression of atherosclerosis.

Although Spain is classified as a low-risk region for CVD mortality (<100 CVD deaths per 100,000) [68], SCA is highly prevalent in middle-aged Spanish men as is the prevalence of traditional cardiovascular risk factors (such as hypertension, dyslipidemia, obesity and smoking). The prevalence of SCA in Spain varies according to the criteria used to define it. In the PESA study, SCA (defined as CAC ≥ 1) was observed in 25 % of asymptomatic employees of the Santander Bank in Madrid [69], whereas in our study, the prevalence of SCA (defined as CAC ≥ 100) was 10.2 %, increasing to 38 % when a CAC ≥ 1 cut-off was used [70]. Regardless of the cut-off used, the high prevalence of SCA remains a significant public health concern, particularly given the potential for many of these cases to progress to major cardiovascular events.

The study of phthalates and their relationship with SCA is of great societal interest due to the public health implications of both. Phthalates are widely used in everyday products and humans are constantly exposed to them from the earliest stages of life. At the same time, SCA can begin early in life and remain asymptomatic for several decades until a major cardiovascular event occurs (myocardial infarction or stroke). Understanding how phthalate exposure contributes to the development of SCA may be useful in developing preventive strategies to reduce the incidence of CVD. The available scientific evidence may also inform public policy aimed at reducing phthalate exposure as a preventive measure for cardiometabolic diseases. Beyond population-level regulations, our findings may also have implications at the individual clinical level. In patients at intermediate cardiovascular risk, reducing phthalate exposure could represent a modifiable environmental factor. A more detailed clinical history could facilitate the identification of potential sources of phthalate exposure. Educational strategies might complement cardiovascular prevention by promoting behaviors that reduce phthalate exposure, such as choosing fragrance-free products, preferring glass containers over plastics, and minimizing ultraprocessed food consumption.

This study has several strengths. First, to our knowledge, it is the first to analyze the association between phthalates and coronary calcification, used as a proxy for SCA. Second, the inclusion of multiple confounders in the analysis minimizes residual confounding. Third, both exposure and outcome were measured using reliable and standardized methods. Fourth, defining SCA as CAC ≥100 improves the clinical interpretability of our findings by identifying participants with a meaningful burden of subclinical atherosclerosis, for whom preventive interventions may be considered. Finally, investigating phthalate exposure is a socially relevant topic, contributing to the growing body of evidence on environmental determinants of CVD.

However, this study has some limitations. First, its cross-sectional design limits the ability to infer causality between phthalates and SCA. Second, the sample population (male workers from a car assembly plant) may limit the generalizability of the findings to other groups, such as women, older adults, or individuals from different socioeconomic or occupational backgrounds. Third, a significant number of participants were excluded due to missing data on urinary creatinine or CAC, which reduced the sample size and the power to detect statistical significance, even if the reported associations were of a certain magnitude for some phthalates. Finally, urinary concentrations of phthalate metabolites reflect short-term exposure, which may not adequately capture long-term exposure, which is more relevant for chronic diseases such as SCA.

In conclusion, this study provides new evidence on the relationship between MEP exposure and SCA, as measured by CAC. The results show that higher urinary MEP concentrations are associated with an increased prevalence of SCA, suggesting that the exposure to phthalates may play a role in the early stages of CVD. Given the widespread exposure to phthalates and their potential impact on the cardiovascular system, these findings highlight the importance of regulatory measures to minimize phthalate exposure. Future longitudinal studies and diverse population-based studies are needed to confirm these findings and to explore the underlying mechanisms. These findings provide a basis for public health initiatives aimed at reducing environmental risk factors and preventing the progression of SCA and thus the incidence of CVD.

## 5 Statement on the use of artificial intelligence

During the preparation of this paper the author(s) used ChatGPT 4o and DeepL Write in order to improve the readability. After using this tool/service, the author(s) reviewed and edited the content accordingly and take(s) full responsibility for the content of the publication.

## CRediT authorship contribution statement

**Diana María Mérida:** Writing – original draft, Visualization, Methodology, Investigation, Formal analysis, Data curation. **Carolina Torrijo-Belanche:** Writing – review & editing. **Belén Moreno-Franco:** Writing – review & editing, Resources, Data curation. **Martín Laclaustra:** Writing – review & editing, Conceptualization. **Jimena Rey-García:** Writing – review & editing. **Sofía Gimeno-Ruiz:** Writing – review & editing. **Ana Bayán-Bravo:** Writing – review & editing. **Pilar Guallar-Castillón:** Writing – review & editing, Validation, Supervision, Resources, Project administration, Methodology, Funding acquisition, Conceptualization.

## Declaration of competing interest

The authors declare that they have no known competing financial interests or personal relationships that could have appeared to influence the work reported in this paper.

## Appendix Supplementary materials

Image, application 1
