# Supplementary material for: Phthalate exposure is associated with subclinical coronary atherosclerosis: The Aragon Workers' Health Study (AWHS)
Source: Am J Prev Cardiol. 2025 Aug 6;23:101072. doi: 10.1016/j.ajpc.2025.101072 (PMC12358661; doi:10.1016/j.ajpc.2025.101072)
Supplement: Supplementary file 1 [file mmc1.docx]

**SUPPLEMENTARY MATERIAL**

**Phthalate exposure is associated with subclinical coronary atherosclerosis:** **The Aragon Workers' Health Study (AWHS)**

This document includes the following:

Supplemental Tables:

- Table S1. Phthalate identification.
- Table S2. Distribution of phthalates in individuals with and without subclinical coronary atherosclerosis.
- Table S3. Sensitivity analysis of the association between phthalates (ng/ml) and subclinical coronary atherosclerosis with creatinine-adjusted models (N=1,119).

Supplemental Figures:

- Figure S1. Predicted margins plots for the association between natural logarithm of phthalates and coronary artery calcium.

**Table S1. Phthalate identification.**

| **Classification** | **Parent compound (abbreviations)** | **Molecular weight (g/mol)** | **CAS registry number** | **Primary metabolite or phthalate monoester (abbreviations)** | **Molecular weight (g/mol)** | **CAS registry number** | **Secondary metabolite or oxidized metabolite (abbreviations)** | **Molecular weight (g/mol)** | **CAS registry number** |
| --- | --- | --- | --- | --- | --- | --- | --- | --- | --- |
| **Low molecular weight (LMW)** | Diethyl phthalate  (DEP) | 222.24 | 84-66-2 | Mono-ethyl phthalate  (MEP) | 194.18 | 2306-33-4 | -- |  |  |
|  | Di-isobutyl phthalate  (DiBP) | 278.34 | 84-69-5 | Mono-isobutyl phthalate (MiBP) | 222.24 | 30833-53-5 | -- |  |  |
|  | Dibutyl phthalate (DBP/DnBP) | 278.34 | 84-74-2 | Mono-n-butyl phthalate (MnBP) | 222.24 | 131-70-4 | -- |  |  |
|  | Benzyl butyl phthalate (BzBP/BBzP) | 312.4 | 85-68-7 | Monobenzyl phthalate (MBzP) | 256.25 | 2528-16-7 | -- |  |  |
| **High molecular weight (HMW)** | Di(2-ethylhexyl) phthalate (DEHP) | 390.6 | 117-81-7 | Mono(2-ethylhexyl) phthalate (MEHP) | 278.34 | 4376-20-9 | Mono(2-ethyl-5-oxohexyl) phthalate (MEOHP/5-oxo-MEHP) | 292.33 | 40321-98-0 |
|  |  |  |  |  |  |  | Mono(2-ethyl-5-carboxypentyl) phthalate  (MECPP/5-cx-MEPP) | 308.33 | 40809-41-4 |
|  |  |  |  |  |  |  | Mono(2-ethyl-5-hydroxyhexyl) phthalate  (MEHHP/ 5-OH-MEHP) | 294.34 | 40321-99-1 |
|  |  |  |  |  |  |  | Mono(2-carboxymethylhexyl) phthalate  (MCMHP/2-cx-MMHP) | 308.33 | 82975-93-7 |
|  | Di-cyclohexyl phthalate (DCHP) | 330.4 | 84-61-7 | Mono-cyclohexyl phthalate (MCHP) | 248.27 | 7517-36-4 |  |  |  |
|  | Dipentyl-phthalate  (DnPP) | 306.4 | 131-18-0 | Mono-n-pentyl phthalate  (MnPeP/ MnPP/MPP) | 236.26 | 24539-56-8 | -- |  |  |
|  | Di-isononyl-phthalate  (DiNP) | 418.6 | 28553-12-0 | Mono-isononyl phthalate (MiNP) | 292.4 | 106610-61-1 | Mono-hydroxy-isononyl phthalate (OH-MiNP) | 308.4 | 936021-98-6 |
|  | Di-iso-decyl phthalate  (DiDP) | 268.26 | 26761-40-0 | Mono-isodecyl phthalate (MiDP) | 306.4 | 31047-64-0 | Mono-carboxy-isodecyl phthalate  (cx-MiDP/ MCDMPHP) | 336.38 | NA |
|  |  |  |  |  |  |  | Mono-hydroxy-isodecyl phthalate (OH-MiDP/ MHDMOP) | 322.40 | NA |
|  | Di-n-octyl phthalate (DnOP) | 390.6 | 117-84-0 | Mono-n-octyl phthalate  (MnOP) | 278.34 | 5393-19-1 | -- |  |  |

**Table S2. Distribution of phthalates in individuals with and without subclinical coronary atherosclerosis.**

| **Phthalate/creatinine**  **(µg/g-creatinine)** | **Non-SCA**  **(n= 1,005)** | **SCA**  **(n= 114)** | **p-value *** |
| --- | --- | --- | --- |
| **MEP** | 99.3 (53.4, 236.3) | 126.1 (61.7, 289.0) | 0.030 |
| **MiBP** | 16.2 (11.5, 23.4) | 17.1 (12.6, 25.5) | 0.252 |
| **MnBP** | 13.4 (8.7, 19.6) | 14.8 (10.5, 20.1) | 0.086 |
| **MBzP** | 5.42 (3.5, 8.97) | 5.55 (3.25, 8.37) | 0.491 |
| **MEOHP** | 6.40 (4.26, 10.09) | 6.40 (4.34, 9.52) | 0.686 |
| **MECPP** | 13.0 (8.60, 20.2) | 13.1 (9.23, 18.6) | 0.942 |
| **MEHHP** | 12.3 (8.05, 19.2) | 12.2 (8.11, 18.36) | 0.976 |
| **MCMHP** | 2.79 (1.98, 4.06) | 2.77 (1.94, 3.94) | 0.539 |
| **OH-MiNP** | 8.18 (5.04, 14.5) | 7.90 (4.92, 17.2) | 0.784 |
| **cx-MiDP** | 1.30 (0.92, 2.05) | 1.32 (1.00, 2.01) | 0.704 |
| **OH-MiDP** | 1.61 (1.04, 2.60) | 1.61 (1.04, 2.60) | 0.463 |
| **ΣDEHP (nmol/ml)** | 0.11 (0.08, 0.18) | 0.11 (0.08, 0.18) | 0.965 |
| Results are presented as medians and interquartile range.  * *p*-values of Mann-Whitney U test  SCA: subclinical coronary atherosclerosis. Non-SCA (CAC < 100) and SCA (CAC ≥ 100). | | | |

**Table S3. Sensitivity analysis of the association between phthalates (ng/ml) and subclinical coronary atherosclerosis with creatinine-adjusted models (N=1,119).**

|  | | Phthalates as continuous | | Phthalates in quartiles | | | | |  |
| --- | --- | --- | --- | --- | --- | --- | --- | --- | --- |
| Phthalate metabolite  (ng/ml) | | OR (95% CI) for  1-unit increase in natural log | p-value | Q1  OR (95% CI) | Q2  OR (95% CI) | Q3  OR (95% CI) | Q4  OR (95% CI) | p-for linear trend |  |
| **MEP** | |  |  |  |  |  |  |  |  |
|  | **Events/n** |  |  | 26/280 | 25/280 | 28/280 | 35/279 |  |  |
|  | **Model 1** | 1.23 (1.05-1.45) * | 0.010 | 1 (Ref.) | 0.99 (0.55-1.76) | 1.15 (0.65-2.05) | 1.50 (0.86-2.63) | 0.123 |  |
|  | **Model 2** | 1.21 (1.01-1.43) * | 0.033 | 1 (Ref.) | 0.96 (0.52-1.77) | 1.02 (0.56-1.87) | 1.32 (0.73-2.40) | 0.343 |  |
| **MiBP** | |  |  |  |  |  |  |  |  |
|  | **Events/n** |  |  | 25/280 | 32/280 | 28/280 | 29/279 |  |  |
|  | **Model 1** | 1.20 (0.88-1.62) | 0.251 | 1 (Ref.) | 1.41 (0.80-2.51) | 1.28 (0.68-2.40) | 1.40 (0.71-2.74) | 0.440 |  |
|  | **Model 2** | 1.23 (0.88-1.72) | 0.219 | 1 (Ref.) | 1.28 (0.69-2.35) | 1.19 (0.62-2.31) | 1.29 (0.63-2.63) | 0.588 |  |
| **MnBP** | |  |  |  |  |  |  |  |  |
|  | **Events/n** |  |  | 27/280 | 25/280 | 27/280 | 35/279 |  |  |
|  | **Model 1** | 1.12 (0.86-1.44) | 0.405 | 1 (Ref.) | 1.01 (0.56-1.82) | 1.15 (0.62-2.10) | 1.63 (0.87-3.03) | 0.103 |  |
|  | **Model 2** | 1.05 (0.78-1.40) | 0.753 | 1 (Ref.) | 0.99 (0.53-1.86) | 0.96 (0.50-1.82) | 1.35 (0.69-2.63) | 0.399 |  |
| **MBzP** | |  |  |  |  |  |  |  |  |
|  | **Events/n** |  |  | 31/280 | 25/280 | 30/280 | 28/279 |  |  |
|  | **Model 1** | 0.87 (0.68-1.11) | 0.266 | 1 (Ref.) | 0.81 (0.46-1.42) | 1.00 (0.57-1.75) | 0.94 (0.52-1.71) | 0.973 |  |
|  | **Model 2** | 0.86 (0.66-1.2) | 0.264 | 1 (Ref.) | 0.74 (0.41-1.35) | 1.00 (0.55-1.81) | 0.96 (0.51-1.81) | 0.852 |  |
| **MEOHP** | |  |  |  |  |  |  |  |  |
|  | **Events/n** |  |  | 30/280 | 30/280 | 27/280 | 27/279 |  |  |
|  | **Model 1** | 0.91 (0.69-1.19) | 0.487 | 1 (Ref.) | 1.03 (0.58-1.81) | 0.92 (0.50-1.68) | 0.93 (0.50-1.72) | 0.730 |  |
|  | **Model 2** | 0.91 (0.67-1.24) | 0.552 | 1 (Ref.) | 1.01 (0.55-1.85) | 0.84 (0.44-1.63) | 0.95 (0.49-1.86) | 0.773 |  |
| **MECPP** | |  |  |  |  |  |  |  |  |
|  | **Events/n** |  |  | 28/280 | 25/280 | 35/280 | 26/279 |  |  |
|  | **Model 1** | 0.95 (0.71-1.25) | 0.695 | 1 (Ref.) | 0.94 (0.52-1.72) | 1.40 (0.78-2.51) | 1.02 (0.54-1.91) | 0.654 |  |
|  | **Model 2** | 0.93 (0.68-1.27) | 0.661 | 1 (Ref.) | 0.95 (0.51-1.81) | 1.59 (0.84-3.02) | 1.00 (0.51-1.99) | 0.675 |  |
| **MEHHP** | |  |  |  |  |  |  |  |  |
|  | **Events/n** |  |  | 30/280 | 28/280 | 26/280 | 30/279 |  |  |
|  | **Model 1** | 0.94 (0.72-1.23) | 0.647 | 1 (Ref.) | 0.96 (0.54-1.71) | 0.90 (0.49-1.66) | 1.07 (0.58-1.97) | 0.867 |  |
|  | **Model 2** | 0.94 (0.69-1.26) | 0.662 | 1 (Ref.) | 0.86 (0.47-1.60) | 0.85 (0.43-1.65) | 1.02 (0.52-2.00) | 0.912 |  |
| **MCMHP** | |  |  |  |  |  |  |  |  |
|  | **Events/n** |  |  | 34/280 | 25/280 | 22/280 | 33/279 |  |  |
|  | **Model 1** | 0.88 (0.65-1.20) | 0.433 | 1 (Ref.) | 0.71 (0.40-1.26) | 0.62 (0.33-1.16) | 0.97 (0.54-1.77) | 0.976 |  |
|  | **Model 2** | 0.96 (0.69-1.34) | 0.830 | 1 (Ref.) | 0.84 (0.46-1.55) | 0.64 (0.33-1.24) | 1.13 (0.59-2.15) | 0.801 |  |
| **OH-MiNP** | |  |  |  |  |  |  |  |  |
|  | **Events/n** |  |  | 32/280 | 28/281 | 19/279 | 35/279 |  |  |
|  | **Model 1** | 1.02 (0.83-1.26) | 0.838 | 1 (Ref.) | 0.87 (0.50-1.51) | 0.58 (0.31-1.07) | 1.13 (0.66-1.95) | 0.852 |  |
|  | **Model 2** | 0.98 (0.78-1.23) | 0.842 | 1 (Ref.) | 0.84 (0.47-1.50) | 0.48 (0.25-0.93) | 1.02 (0.57-1.82) | 0.772 |  |
| **cx-MiDP** | |  |  |  |  |  |  |  |  |
|  | **Events/n** |  |  | 26/280 | 29/281 | 33/279 | 26/279 |  |  |
|  | **Model 1** | 1.02 (0.78-1.34) | 0.858 | 1 (Ref.) | 1.18 (0.67-2.09) | 1.41 (0.79-2.50) | 1.08 (0.59-1.99) | 0.697 |  |
|  | **Model 2** | 1.04 (0.77-1.40) | 0.809 | 1 (Ref.) | 1.15 (0.63-2.10) | 1.39 (0.75-2.55) | 1.12 (0.59-2.15) | 0.614 |  |
| **OH-MiDP** | |  |  |  |  |  |  |  |  |
|  | **Events/n** |  |  | 34/280 | 23/280 | 29/280 | 28/279 |  |  |
|  | **Model 1** | 0.91 (0.71-1.18) | 0.486 | 1 (Ref.) | 0.65 (0.37-1.16) | 0.84 (0.48-1.48) | 0.82 (0.46-1.46) | 0.716 |  |
|  | **Model 2** | 0.87 (0.66-1.15) | 0.337 | 1 (Ref.) | 0.64 (0.35-1.17) | 0.75 (0.41-1.36) | 0.77 (0.41-1.44) | 0.538 |  |
| **ΣDEHP (nmol/ml)** | |  |  |  |  |  |  |  |  |
|  | **Events/n** |  |  | 31/280 | 26/280 | 24/280 | 33/279 |  |  |
|  | **Model 1** | 0.93 (0.70-1.24) | 0.612 | 1 (Ref.) | 0.86 (0.47-1.54) | 0.79 (0.43-1.47) | 1.14 (0.62-2.09) | 0.640 |  |
|  | **Model 2** | 0.92 (0.67-1.27) | 0.625 | 1 (Ref.) | 0.83 (0.44-1.56) | 0.80 (0.41-1.56) | 1.12 (0.58-2.18) | 0.662 |  |
| * *p* < 0.05, ** *p* < 0.01. OR: Odds Ratio; CI: confidence interval.  Model 1: adjusted for urinary creatinine (mg/dL). Model 2: as in model 1 and additionally adjusted for age (continuous), BMI (<25, ≥25 - <30, ≥30), smoking status (non-smoker, former, current), alcohol consumption (g/day), work type (office/manual work), work turn (morning/afternoon, morning/afternoon/night, central and night), hypertension (no/yes), dyslipidemia (no/yes), diabetes (no/yes), physical activity (METs-h/week), total energy intake (Kcal/day). | | | | | | | | |  |

**Table S4. Association between phthalates and subclinical coronary atherosclerosis after excluding participants with diabetes (N=1,059).**

|  | | Phthalates as continuous | | Phthalates in quartiles | | | | |
| --- | --- | --- | --- | --- | --- | --- | --- | --- |
| Phthalate metabolite  (µg/g-creatinine) | | OR (95% CI) for  1-unit increase in natural log | p-value | Q1  OR (95% CI) | Q2  OR (95% CI) | Q3  OR (95% CI) | Q4  OR (95% CI) | p-for linear trend |
| **MEP** | |  |  |  |  |  |  |  |
|  | **Events/n** |  |  | 22/271 | 22/266 | 23/263 | 28/259 |  |
|  | **Crude model** | 1.20 (1.01-1.42) * | 0.044 | Ref. | 1.02 (0.55-1.89) | 1.08 (0.59-2.00) | 1.37 (0.76-2.47) | 0.280 |
|  | **Adjusted model** | 1.20 (1.00-1.44) * | 0.048 | Ref. | 1.07 (0.56-2.02) | 1.11 (0.59-2.09) | 1.31 (0.71-2.42) | 0.380 |
| **MiBP** | |  |  |  |  |  |  |  |
|  | **Events/n** |  |  | 20/266 | 25/266 | 22/264 | 28/263 |  |
|  | **Crude model** | 1.19 (0.85-1.66) | 0.306 | Ref. | 1.28 (0.69-2.36) | 1.12 (0.59-2.10) | 1.47 (0.80-2.67) | 0.291 |
|  | **Adjusted model** | 1.22 (0.86-1.73) | 0.259 | Ref. | 1.47 (0.77-2.81) | 1.17 (0.60-2.26) | 1.48 (0.79-2.78) | 0.355 |
| **MnBP** | |  |  |  |  |  |  |  |
|  | **Events/n** |  |  | 16/267 | 28/266 | 26/262 | 25/264 |  |
|  | **Crude model** | 1.06 (0.80-1.42) | 0.675 | Ref. | 1.85 (0.97-3.50) | 1.73 (0.90-3.30) | 1.64 (0.85-3.15) | 0.210 |
|  | **Adjusted model** | 1.02 (0.74-1.40) | 0.908 | Ref. | 1.93 (1.00-3.75) | 1.75 (0.89-3.43) | 1.53 (0.78-3.03) | 0.342 |
| **MBzP** | |  |  |  |  |  |  |  |
|  | **Events/n** |  |  | 28/266 | 18/264 | 30/266 | 19/263 |  |
|  | **Crude model** | 0.79 (0.59-1.04) | 0.094 | Ref. | 0.62 (0.34-1.15) | 1.08 (0.63-1.86) | 0.66 (0.36-1.22) | 0.490 |
|  | **Adjusted model** | 0.79 (0.59-1.07) | 0.135 | Ref. | 0.61 (0.32-1.16) | 1.19 (0.67-2.11) | 0.71 (0.37-1.34) | 0.722 |
| **MEOHP** | |  |  |  |  |  |  |  |
|  | **Events/n** |  |  | 24/267 | 26/262 | 25/261 | 20/269 |  |
|  | **Crude model** | 0.85 (0.62-1.16) | 0.303 | Ref. | 1.12 (0.62-2.00) | 1.07 (0.60-1.93) | 0.81 (0.44-1.51) | 0.520 |
|  | **Adjusted model** | 0.88 (0.63-1.22) | 0.447 | Ref. | 1.18 (0.64-2.18) | 1.16 (0.62-2.15) | 0.88 (0.46-1.69) | 0.715 |
| **MECPP** | |  |  |  |  |  |  |  |
|  | **Events/n** |  |  | 20/245 | 30/268 | 28/261 | 17/265 |  |
|  | **Crude model** | 0.89 (0.65-1.22) | 0.480 | Ref. | 1.54 (0.85-2.79) | 1.47 (0.81-2.69) | 0.84 (0.43-1.64) | 0.618 |
|  | **Adjusted model** | 0.91 (0.65-1.28) | 0.590 | Ref. | 1.57 (0.84-2.93) | 1.67 (0.89-3.13) | 0.87 (0.43-1.76) | 0.789 |
| **MEHHP** | |  |  |  |  |  |  |  |
|  | **Events/n** |  |  | 27/267 | 23/262 | 22/261 | 23/269 |  |
|  | **Crude model** | 0.88 (0.65-1.19) | 0.420 | Ref. | 0.86 (0.48-1.53) | 0.82 (0.45-1.48) | 0.83 (0.46-1.49) | 0.520 |
|  | **Adjusted model** | 0.90 (0.65-1.25) | 0.542 | Ref. | 0.82 (0.45-1.51) | 0.79 (0.43-1.47) | 0.89 (0.48-1.65) | 0.693 |
| **MCMHP** | |  |  |  |  |  |  |  |
|  | **Events/n** |  |  | 27/261 | 26/271 | 21/263 | 21/264 |  |
|  | **Crude model** | 0.81 (0.57-1.15) | 0.246 | Ref. | 0.92 (0.52-1.62) | 0.75 (0.41-1.37) | 0.75 (0.41-1.36) | 0.266 |
|  | **Adjusted model** | 0.90 (0.62-1.30) | 0.560 | Ref. | 0.96 (0.53-1.75) | 0.85 (0.45-1.59) | 0.83 (0.44-1.56) | 0.507 |
| **OH-MiNP** | |  |  |  |  |  |  |  |
|  | **Events/n** |  |  | 27/270 | 26/271 | 17/255 | 25/263 |  |
|  | **Crude model** | 0.99 (0.79-1.25) | 0.957 | Ref. | 0.96 (0.54-1.68) | 0.64 (0.34-1.21) | 0.94 (0.53-1.67) | 0.575 |
|  | **Adjusted model** | 0.96 (0.76-1.22) | 0.759 | Ref. | 1.00 (0.55-1.80) | 0.58 (0.30-1.12) | 0.92 (0.51-1.69) | 0.469 |
| **cx-MiDP** | |  |  |  |  |  |  |  |
|  | **Events/n** |  |  | 23/270 | 27/262 | 27/270 | 18/257 |  |
|  | **Crude model** | 0.89 (0.65-1.21) | 0.446 | Ref. | 1.23 (0.69-2.21) | 1.19 (0.67-2.14) | 0.81 (0.43-1.54) | 0.556 |
|  | **Adjusted model** | 0.90 (0.64-1.27) | 0.552 | Ref. | 1.24 (0.67-2.27) | 1.13 (0.61-2.09) | 0.84 (0.43-1.65) | 0.608 |
| **OH-MiDP** | |  |  |  |  |  |  |  |
|  | **Events/n** |  |  | 32/265 | 22/270 | 23/266 | 18/258 |  |
|  | **Crude model** | 0.75 (0.56-1.02) | 0.063 | Ref. | 0.65 (0.36-1.14) | 0.69 (0.39-1.21) | 0.55 (0.30-1.00) | 0.062 |
|  | **Adjusted model** | 0.75 (0.54-1.03) | 0.076 | Ref. | 0.59 (0.32-1.08) | 0.65 (0.36-1.18) | 0.53 (0.28-1.02) | 0.075 |
| **ΣDEHP (µmol/g-creatinine)** | |  |  |  |  |  |  |  |
|  | **Events/n** |  |  | 25/266 | 22/267 | 28/259 | 20/267 |  |
|  | **Crude model** | 0.87 (0.63-1.20) | 0.404 | Ref. | 0.87 (0.48-1.58) | 1.17 (0.66-2.06) | 0.78 (0.42-1.44) | 0.682 |
|  | **Adjusted model** | 0.89 (0.63-1.27) | 0.531 | Ref. | 0.94 (0.50-1.77) | 1.22 (0.69-2.21) | 0.87 (0.46-1.67) | 0.917 |
| * p < 0.05, ** p < 0.01. OR: Odds Ratio; CI: confidence interval.  Model 1: adjusted for urinary creatinine (mg/dL). Model 2: as in model 1 and additionally adjusted for age (continuous), BMI (<25, ≥25 - <30, ≥30), smoking status (non-smoker, former, current), alcohol consumption (g/day), work type (office/manual work), work turn (morning/afternoon, morning/afternoon/night, central and night), hypertension (no/yes), dyslipidemia (no/yes), physical activity (METs-h/week), total energy intake (Kcal/day), and fasting glucose levels (continuous). | | | | | | | | |

| **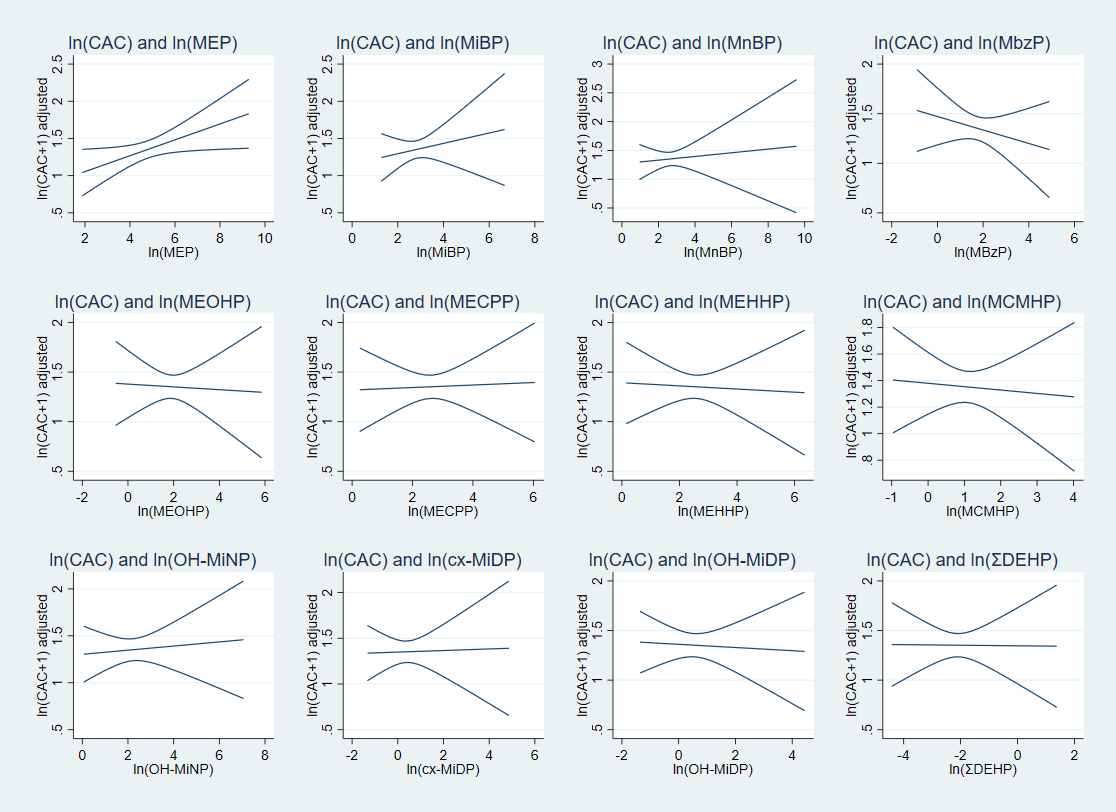** |
| --- |
| **Figure S1. Predicted margins plots for the association between natural logarithm of phthalates and coronary artery calcium.** Predicted margins plots show the adjusted associations between natural log-transformed (ln) phthalate concentrations and coronary artery calcium (CAC). Predicted values of ln(CAC+1) were derived from separate linear regression models for each phthalate, adjusting for all covariates in the fully adjusted model. Predictions were calculated across the observed range of ln(phthalate) values (in 0.2-unit increments), holding other covariates at their means. Both CAC and phthalate levels were log-transformed to reduce skewness. Linear trends are shown with 95% confidence intervals. |
